# Evaluation of a Caregiver-Completed Questionnaire for Assessing Early Childhood Caries Risk: A Cross-Sectional Study

**DOI:** 10.7759/cureus.112685

**Published:** 2026-07-14

**Authors:** Divya Sree Gunnam, Akshitha Ravikumar, Akshat A Chokshi, Sameer Chauhan, Gayathri Sreedhar, Prashanth Kumar B

**Affiliations:** 1 Department of Public Health, Master of Public Health, University of New Haven, West Haven, USA; 2 Department of Public Health Dentistry, Panineeya Institute of Dental Sciences and Research Centre, Hyderabad, IND; 3 Department of Dentistry, Taunton Village Dental, Oshawa, CAN; 4 Department of Prosthodontics, K. M. Shah Dental College and Hospital, Sumandeep Vidyapeeth (Deemed to be University), Vadodara, IND; 5 Department of Public Health Dentistry, Azeezia College of Dental Sciences and Research, Adichanalloor, IND; 6 Department of Pedodontics and Preventive Dentistry, Tirumala Institute of Dental Sciences and Research Centre, Nizamabad, IND

**Keywords:** caregivers, dental caries risk assessment, early childhood caries, oral health screening, questionnaires

## Abstract

Introduction

Early childhood caries (ECC) is one of the most prevalent chronic diseases affecting preschool children worldwide and can adversely affect oral health, nutrition, growth, and quality of life. Early identification of children at risk is essential for implementing timely preventive interventions. Caregiver-completed screening tools may offer a simple and cost-effective approach to identifying children who require further dental evaluation. Therefore, the present study aimed to evaluate the diagnostic performance of a self-developed, caregiver-completed questionnaire for identifying ECC among preschool children, using clinical examination as the reference standard.

Materials and methods

A cross-sectional study was conducted among 150 caregiver-child dyads, including children aged 3-6 years. Caregivers completed a self-developed, 15-item questionnaire covering feeding practices, dietary habits, oral hygiene practices, dental history, and caregiver-related factors. Content validation was performed by an expert panel, followed by pilot testing and reliability assessment. All children subsequently underwent a clinical examination for the diagnosis of ECC. Internal consistency was evaluated using Cronbach’s alpha. Receiver operating characteristic (ROC) curve analysis was performed to determine the optimal questionnaire cutoff score. Sensitivity, specificity, predictive values, likelihood ratios, area under the curve (AUC), and Cohen’s kappa were calculated.

Results

Clinical examination identified ECC in 90 (60.0%) children, while 60 (40.0%) children did not have ECC. Questionnaire scores were significantly higher among children with ECC (19.4 ± 4.6) than among those without ECC (10.2 ± 3.8; p < 0.001). The questionnaire demonstrated good internal consistency, with an overall Cronbach’s alpha of 0.86. ROC curve analysis revealed an AUC of 0.884 (95% CI: 0.830-0.938). The optimal cutoff score was ≥15, yielding a sensitivity of 82.2%, specificity of 86.7%, positive predictive value of 90.2%, and negative predictive value of 76.5%. The positive and negative likelihood ratios were 6.17 and 0.205, respectively. Agreement analysis demonstrated an observed agreement of 84.0% and a Cohen’s kappa coefficient of 0.672, indicating substantial agreement with the clinical diagnosis.

Conclusion

The caregiver-completed ECC questionnaire demonstrated good reliability, substantial agreement with clinical examination findings, and good diagnostic performance as a screening tool. The questionnaire may serve as a simple, non-invasive, and cost-effective tool for the early identification of children with or at risk of ECC and may facilitate timely preventive interventions in community and clinical settings.

## Introduction

Early childhood caries (ECC) is one of the most prevalent chronic diseases affecting preschool children worldwide and remains a significant public health concern. It is characterized by the presence of one or more decayed, missing, or filled tooth surfaces in any primary tooth in a child younger than six years of age [1]. ECC can adversely affect a child’s oral health, nutrition, growth, quality of life, and overall well-being [2]. Despite advances in preventive dentistry, a substantial proportion of preschool children continue to experience untreated carious lesions, particularly in low- and middle-income countries [3].

The development of ECC is multifactorial and involves complex interactions among dietary habits, oral hygiene practices, feeding behaviors, socioeconomic factors, parental awareness, and microbial influences [2]. Early identification of children at risk is essential for implementing timely preventive interventions and reducing the burden of disease. Conventional diagnosis of ECC relies on clinical examinations performed by dental professionals; however, access to dental care may be limited because of geographical, financial, or logistical barriers. Consequently, simple and reliable screening tools that can be used by caregivers have gained increasing attention [4,5].

Caregiver-completed questionnaires represent a potentially cost-effective and accessible approach to identifying children at risk of ECC [6,7]. Such tools can capture information on feeding practices, sugar consumption, oral hygiene habits, dental history, and family-related risk factors. If demonstrated to be reliable and diagnostically accurate, caregiver-completed questionnaires could facilitate early referral and preventive care, particularly in community-based settings and underserved populations.

Therefore, the present study aimed to evaluate the diagnostic accuracy of a caregiver-completed ECC risk questionnaire for detecting ECC among preschool children, using clinical examination as the reference standard. The specific objectives were to assess the internal consistency of the questionnaire; determine the optimal diagnostic cutoff score; evaluate its sensitivity, specificity, predictive values, likelihood ratios, and area under the receiver operating characteristic (ROC) curve; and assess the agreement between questionnaire-based classification and the clinical diagnosis of ECC.

## Materials and methods

Study design and setting

A cross-sectional study was conducted among preschool children aged 3-6 years and their primary caregivers attending the Department of Public Health Dentistry at Azeezia College of Dental Sciences and Research, Kollam, India, between June and November 2022. Ethical approval was obtained from the Institutional Ethics Committee (AEC/2022/29) before the commencement of the study. Written informed consent was obtained from all participating caregivers before enrollment.

Sample size calculation

The sample size was estimated using G*Power software, version 3.1.9.7 (Heinrich Heine University, Düsseldorf, Germany). Based on the findings of a previous study evaluating caregiver-based caries screening tools, an effect size of 0.50 was considered. With a 95% confidence level, a statistical power of 80%, and a significance level of 0.05, the minimum required sample size was calculated to be 134 participants [8]. To compensate for potential exclusions and incomplete responses and to improve the precision of the diagnostic estimates, a total of 150 caregiver-child dyads were included in the study.

Participant selection

Eligible caregiver-child dyads were recruited using consecutive sampling. Children aged 3-6 years who were accompanied by a parent or primary caregiver who was able to understand and complete the questionnaire were considered eligible for participation. Children with systemic diseases affecting oral health, developmental dental anomalies, or special healthcare needs that could influence oral examination findings were excluded. Caregiver-child dyads were also excluded if the caregivers were unwilling to participate.

Development of the questionnaire

A caregiver-completed questionnaire was developed by the investigators following an extensive review of the literature on established risk factors associated with ECC. The initial item pool was generated by DSG, AR, SC, PKB, and GS, while questionnaire refinement and content review were performed collaboratively by all investigators. Potential items were generated based on evidence from published ECC risk assessment tools, clinical guidelines, and epidemiological studies. Through a series of discussions and consensus meetings among the authors, the most relevant items were selected and organized into five domains: breastfeeding and nighttime bottle-feeding practices; consumption of sugary drinks and snacks; oral hygiene practices; dental history and visible oral problems; and family- or caregiver-related factors. The preliminary questionnaire consisted of 15 items formulated in simple and easily understandable language to facilitate accurate caregiver responses (Appendix 1). Responses were scored according to the presence or absence of established ECC risk factors, and a cumulative risk score was calculated for each participant. Each response indicating the presence of an established ECC risk factor was assigned 1 point, whereas the absence of the risk factor was assigned 0 points. The total questionnaire score ranged from 0 to 15, with higher scores indicating a greater risk of ECC. The questionnaire subsequently underwent expert content validation, pilot testing, and reliability assessment before its application in the main study.

Content validity assessment

The questionnaire was initially developed by the study investigators following a comprehensive review of the literature on risk factors associated with early childhood caries. Subsequently, the draft questionnaire was evaluated for content validity by a panel of seven independent experts comprising pediatric dentists, public health dentists, and dental researchers with experience in questionnaire development and ECC research. The experts independently assessed each item for relevance, clarity, simplicity, and comprehensiveness using a four-point rating scale. Their recommendations were reviewed by the investigators and incorporated into subsequent revisions of the questionnaire. Items considered ambiguous, redundant, or inadequately representative of the construct were modified or removed. The final version of the questionnaire was prepared after consensus was reached among the expert panel members, confirming that the instrument adequately represented the domains relevant to ECC risk assessment.

Pilot testing

Before the main study, pilot testing was conducted among 20 caregiver-child dyads who fulfilled the eligibility criteria but were not included in the final analysis. The purpose of the pilot study was to evaluate the comprehensibility, acceptability, and feasibility of administering the questionnaire. Caregivers were encouraged to provide feedback regarding the wording and interpretation of the questions and the ease of completing the questionnaire. Minor modifications were made to improve clarity and readability based on participant feedback. The average time required to complete the questionnaire was approximately 5-7 minutes, indicating good feasibility for routine clinical use.

Reliability assessment

The internal consistency of the questionnaire was assessed using Cronbach’s alpha coefficient. The reliability analysis demonstrated good internal consistency for the overall questionnaire, with a Cronbach’s alpha value of 0.86. Domain-specific alpha coefficients ranged from 0.71 to 0.82, indicating acceptable to good internal consistency across the individual domains. These findings suggest that the questionnaire items consistently assessed ECC risk and support the instrument’s suitability for further evaluation in the study population.

Clinical examination

Following completion of the questionnaire, all children underwent a standardized clinical oral examination performed by a calibrated examiner (GS) under adequate illumination. ECC was diagnosed according to the criteria established by the American Academy of Pediatric Dentistry (AAPD), which defines ECC as the presence of one or more decayed tooth surfaces, including non-cavitated or cavitated lesions; missing tooth surfaces due to caries; or filled tooth surfaces in any primary tooth in a child 71 months of age or younger [9]. The dmft/deft (decayed, missing/extracted, and filled teeth) indices were also recorded for each participant [10]. Children presenting with at least one decayed, missing due to caries, or filled primary tooth were classified as ECC-positive, whereas those with no evidence of decayed, missing due to caries, or filled primary teeth were classified as ECC-negative. The clinical examination findings served as the reference standard against which the questionnaire-based classifications were compared to evaluate the screening performance of the caregiver-completed questionnaire.

Examiner calibration

To ensure consistency in the clinical diagnosis, examiner calibration was conducted before the study. Ten children were re-examined after a one-week interval, and intra-examiner reliability was assessed using Cohen’s kappa statistic and the intraclass correlation coefficient. A kappa value of 0.88 for ECC diagnosis and an intraclass correlation coefficient of 0.91 were obtained, indicating excellent reproducibility and agreement.

Data collection procedure

Eligible caregivers completed the questionnaire independently before the clinical examination of their children. Questionnaire responses were reviewed for completeness, and total risk scores were calculated. Clinical findings were recorded separately by an examiner who was blinded to the questionnaire scores. Questionnaire-based classifications and clinical diagnoses were subsequently compared to determine the screening performance of the instrument.

Statistical analysis

Statistical analysis was performed using SPSS software, version 26.0 (IBM Corp., Armonk, NY, USA). Descriptive statistics were expressed as frequencies and percentages for categorical variables and as mean ± SD for continuous variables. Associations between categorical variables were evaluated using the chi-square test, while differences in mean questionnaire scores between the groups with and without ECC were analyzed were analyzed using the independent-samples t-test, with equal variances assumed. The internal consistency of the questionnaire was assessed using Cronbach’s alpha coefficient. ROC curve analysis was performed to determine the optimal cutoff score using Youden’s index. Sensitivity, specificity, positive predictive value, negative predictive value, likelihood ratios, and the area under the ROC curve were calculated to assess screening performance. Agreement between questionnaire-based classification and clinical diagnosis was evaluated using Cohen’s kappa statistic. Statistical significance was set at p < 0.05.

## Results

In total, 150 participants were included in this study. Clinical examination identified ECC in 90 (60.0%) children, while 60 (40.0%) children were free of ECC. As shown in Table 1, among children with ECC, the largest proportion belonged to the 5-year-old age group, followed by the 4-year-old and 6-year-old age groups, and then the 3-year-old age group. The age distribution did not differ significantly between children with and without ECC (χ² = 1.41, df = 3, p = 0.702). Regarding sex distribution (Table 1), 50 male children (55.6%) were in the ECC group and 28 (46.7%) were in the non-ECC group, whereas female children accounted for 40 (44.4%) and 32 (53.3%) children, respectively. No significant association was observed between sex and ECC status (p = 0.286).

**Table 1:** Demographic characteristics and questionnaire scores according to early childhood caries status. ECC: Early childhood caries; df: degrees of freedom. Values are presented as n (%) or mean ± SD. p-values were calculated using the chi-square test for categorical variables and the independent-samples t-test for continuous variables. A p-value < 0.05 was considered statistically significant. *Indicates a statistically significant difference.

Variable	Category	ECC present (n = 90)	ECC absent (n = 60)	Test statistic (df)	p-value
Age group, n (%)	3 years	18 (20.0%)	8 (13.3%)	χ² = 1.41 (3)	0.702
	4 years	22 (24.4%)	14 (23.3%)		
	5 years	28 (31.1%)	20 (33.3%)		
	6 years	22 (24.4%)	18 (30.0%)		
Sex, n (%)	Male	50 (55.6%)	28 (46.7%)	χ² = 1.41 (1)	0.286
	Female	40 (44.4%)	32 (53.3%)		
Caregiver education, n (%)	No formal schooling	12 (13.3%)	3 (5.0%)	χ² = 10.70 (3)	0.013*
	Primary school	30 (33.3%)	10 (16.7%)		
	Secondary school	32 (35.6%)	27 (45.0%)		
	Higher education	16 (17.8%)	20 (33.3%)		
Relationship of respondent, n (%)	Mother	68 (75.6%)	44 (73.3%)	χ² = 0.22 (3)	0.975
	Father	14 (15.6%)	10 (16.7%)		
	Grandparent	6 (6.7%)	4 (6.7%)		
	Other	2 (2.2%)	2 (3.3%)		
Questionnaire score	Mean ± SD	19.4 ± 4.6	10.2 ± 3.8	t = 12.84 (148)	< 0.001*

Caregiver education level was significantly associated with ECC status (Table 1). Caregivers with no formal schooling were more common among children with ECC than among those without ECC. In contrast, higher education levels were more frequently observed among caregivers of children without ECC than among those with ECC (p = 0.013). Questionnaire scores were significantly higher among children with ECC (19.4 ± 4.6) than among those without ECC (10.2 ± 3.8; p < 0.001) (Table 1). The questionnaire demonstrated good internal consistency (Table 2). Cronbach’s alpha values ranged from 0.71 to 0.82 for the individual domains, while the overall 15-item questionnaire demonstrated a Cronbach’s alpha of 0.86.

**Table 2:** Internal consistency of the early childhood caries risk questionnaire. ECC: Early childhood caries. Cronbach’s alpha (α) was used to assess internal consistency reliability. An α value ≥ 0.70 was considered acceptable, and an α value ≥ 0.80 was considered indicative of good reliability.

Domain	No. of items	Cronbach’s alpha (α)	Interpretation
Breastfeeding and nighttime bottle-feeding	2	0.74	Acceptable
Consumption of sugary drinks and snacks	3	0.78	Acceptable
Oral hygiene practices	4	0.82	Good
Dental history and visible oral problems	4	0.77	Acceptable
Family- and caregiver-related factors	2	0.71	Acceptable
Overall questionnaire	15	0.86	Good

ROC curve analysis (Table 3) demonstrated good discriminatory ability, with an AUC of 0.884 (95% CI: 0.830-0.938). The optimal cutoff score was ≥15, corresponding to a sensitivity of 82.2% and a specificity of 86.7%.

**Table 3:** Sensitivity, specificity, predictive values, and Youden’s index at different questionnaire cutoff scores. PPV: Positive predictive value; NPV: Negative predictive value; ROC: Receiver operating characteristic. Values are presented as percentages unless otherwise specified. *Indicates the optimal cutoff score determined using the maximum Youden’s index.

Cutoff score	Sensitivity	Specificity	PPV	NPV	Youden’s J
≥10	95.60%	46.70%	72.90%	87.50%	0.423
≥12	92.20%	61.70%	78.30%	84.10%	0.539
≥14	87.80%	80.00%	86.80%	81.40%	0.678
≥15*	82.20%	86.70%	90.20%	76.50%	0.689
≥17	75.60%	90.00%	91.90%	71.10%	0.656
≥19	62.20%	93.30%	93.30%	62.20%	0.555
≥21	48.90%	96.70%	95.70%	55.80%	0.456

Using the optimal cutoff score (Table 4), 82 (54.7%) children were classified as questionnaire-positive and 68 (45.3%) as questionnaire-negative. Among the questionnaire-positive children, 74 (90.2%) were true positives and eight (9.8%) were false positives. Among the questionnaire-negative children, 52 (76.5%) were true negatives and 16 (23.5%) were false negatives.

**Table 4:** Comparison of questionnaire-based classification and clinical diagnosis of early childhood caries TP: True positive; FP: False positive; FN: False negative; TN: True negative; ECC: Early childhood caries. Questionnaire-positive classification was defined as a score ≥15. Clinical examination served as the reference standard.

Questionnaire classification	ECC present (clinical examination positive)	ECC absent (clinical examination negative)	Total
Questionnaire-positive (score ≥15)	74 (TP)	8 (FP)	82
Questionnaire-negative (score <15)	16 (FN)	52 (TN)	68
Total	90	60	150

As presented in Table 5, the questionnaire demonstrated a sensitivity of 82.2% (95% CI: 72.9%-89.4%), specificity of 86.7% (95% CI: 75.4%-93.9%), positive predictive value of 90.2%, negative predictive value of 76.5%, positive likelihood ratio of 6.18, and negative likelihood ratio of 0.205.

**Table 5:** Diagnostic performance of the caregiver-completed early childhood caries risk questionnaire. Values are presented as estimates with 95% CIs, where applicable.

Diagnostic metric	Estimate	95% CI
Sensitivity	82.20%	72.9%-89.4%
Specificity	86.70%	75.4%-93.9%
Positive predictive value (PPV)	90.20%	81.7%-95.6%
Negative predictive value (NPV)	76.50%	64.6%-85.7%
Positive likelihood ratio (LR+)	6.17	3.21-11.89
Negative likelihood ratio (LR-)	0.205	0.134-0.315
Youden’s index (J)	0.689	-
Optimal cutoff score	≥15	-
Area under the receiver operating characteristic curve (AUC)	0.884	0.830-0.938

**Figure 1:**
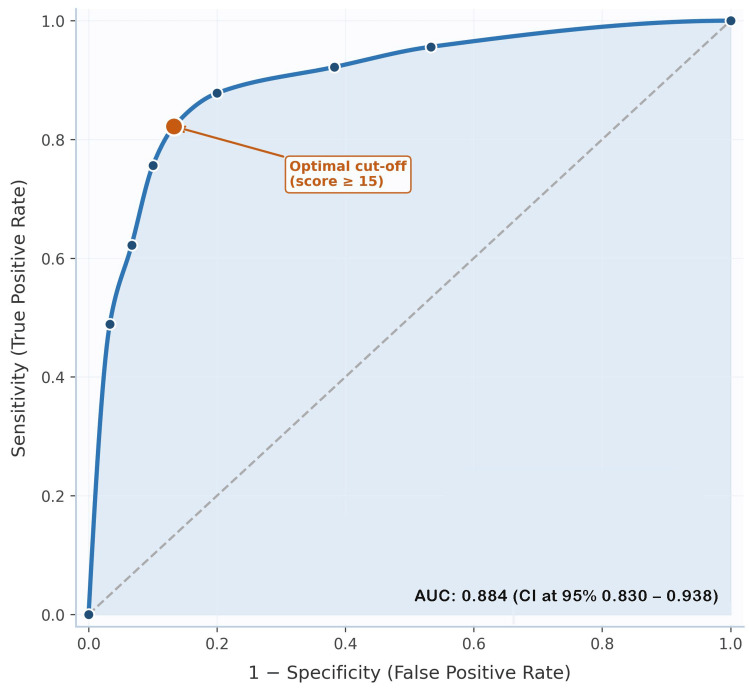
Receiver operating characteristic (ROC) curve of the questionnaire score for predicting the presence of early childhood caries. The area under the curve (AUC) was 0.884 (95% CI: 0.830-0.938), indicating good discriminatory accuracy.

Youden’s index was 0.689, and the area under the ROC curve was 0.884 (95% CI: 0.830-0.938), confirming good overall diagnostic accuracy (Figure 1).

The agreement analysis revealed 84.0% agreement between the questionnaire-based classification and the clinical diagnosis (Table 6). Cohen’s kappa coefficient was 0.672 (95% CI: 0.554-0.790), indicating substantial agreement beyond chance. The agreement was statistically significant (p < 0.001).

**Table 6:** Agreement between questionnaire-based classification and the clinical diagnosis of early childhood caries. Cohen’s kappa (κ) was used to assess agreement beyond chance. The p-value was calculated using a two-tailed z-test. *p < 0.05 was considered statistically significant.

Agreement measure	Value
Observed agreement	84.00%
Cohen’s kappa (κ)	0.674
95% CI for κ	0.555-0.793
p-value	<0.001*

**Figure 2:**
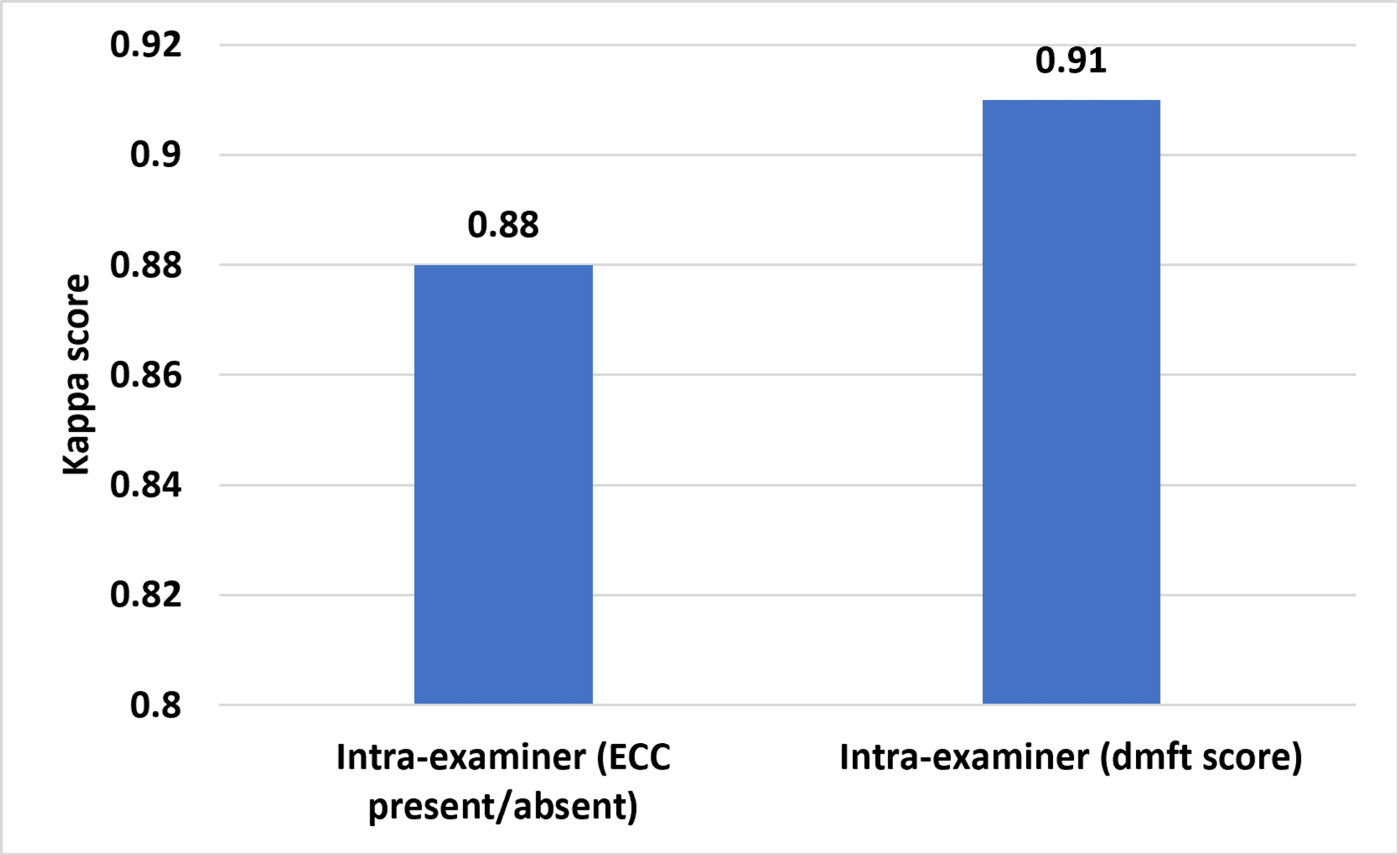
Intra-examiner agreement for ECC diagnosis using Cohen’s kappa and for dmft scoring using the intraclass correlation coefficient. Kappa values >0.80 indicate almost perfect agreement. The kappa value of 0.88 and the intraclass correlation coefficient of 0.91 indicate excellent intra-examiner reproducibility. ECC: Early childhood caries.

As shown in Figure 2, the intra-examiner calibration demonstrated excellent reliability. Cohen’s kappa coefficient for ECC diagnosis was 0.88, while the intraclass correlation coefficient for dmft scoring was 0.91, indicating a high level of reproducibility and consistency in the clinical assessments.

## Discussion

ECC remains one of the most common chronic diseases affecting preschool children worldwide and continues to pose significant challenges for early detection and prevention [1]. This study evaluated the performance of a self-developed, caregiver-completed ECC risk questionnaire and demonstrated that the instrument possessed good reliability, substantial agreement with the clinical diagnosis, and excellent overall screening performance. These findings support the potential utility of caregiver-based risk assessment tools as adjunctive methods for the early identification of children with or at risk of ECC.

In the present study, the prevalence of ECC was 60.0%, indicating a considerable disease burden among preschool children. Similarly, high prevalence rates have been reported in developing countries, where socioeconomic factors, dietary practices, inadequate oral hygiene, and limited access to preventive dental care contribute to increased disease occurrence [4,11]. The significant association observed between caregiver education level and ECC status is consistent with previous studies that identified parental education as an important determinant of children’s oral health [12,13]. Caregivers with lower educational attainment may have reduced awareness of appropriate feeding practices, oral hygiene measures, and the importance of preventive dental visits, thereby increasing the risk of ECC.

The questionnaire demonstrated good internal consistency, with an overall Cronbach’s alpha of 0.86. This finding indicates that the questionnaire items reliably measured related aspects of ECC risk. The acceptable-to-good internal consistency observed across the individual domains further supports the adequacy of the questionnaire’s structure and content. Custodio-Lumsden CL et al. [14] validated the MySmileBuddy ECC risk assessment tool and reported significant associations between questionnaire-based risk scores and established caries risk indicators, such as salivary mutans streptococci levels and visible dental plaque.

One of the most important findings of the present study was the excellent diagnostic performance of the questionnaire. The area under the ROC curve was 0.884, indicating excellent discriminatory ability. The optimal cutoff score of ≥15 provided a sensitivity of 82.2% and a specificity of 86.7%, suggesting that the questionnaire was capable of correctly identifying most children with ECC while minimizing false-positive results. These findings are comparable to those of previous caregiver-based screening approaches. Saikia A et al. [8] developed MAAC charts as a parent-friendly screening tool and reported that caregiver-assisted identification methods could facilitate the early recognition of ECC and improve referral for professional evaluation. Similarly, studies evaluating caregiver-reported oral health questionnaires have demonstrated that parental observations and risk factor assessments can effectively identify children requiring further dental examinations [6,7].

The positive predictive value of 90.2% observed in the present study indicates that most children classified as high-risk by the questionnaire were confirmed to have ECC on clinical examination. Furthermore, the positive likelihood ratio of 6.18 and the negative likelihood ratio of 0.205 suggest good discriminatory capacity and clinical usefulness. These findings indicate that the questionnaire may be valuable as a preliminary screening instrument in community settings, schools, childcare centers, and primary healthcare facilities, where routine dental examinations may not always be available.

The agreement analysis revealed a Cohen’s kappa coefficient of 0.672, indicating substantial agreement between the questionnaire-based classification and the clinical diagnosis. The observed agreement of 84.0% further supported the validity of the questionnaire. Similar levels of agreement have been reported in studies evaluating parent-reported oral health assessments and caries risk-screening tools [15,16]. The substantial agreement obtained in the present study suggests that caregivers can provide meaningful information regarding the behaviors and risk factors associated with ECC when appropriately guided through a structured questionnaire.

A notable strength of the present study is the comprehensive psychometric and diagnostic evaluation of the caregiver-completed questionnaire. While previous caregiver-based ECC screening tools, including the MAAC charts developed by Saikia A et al. [8] and the MySmileBuddy risk assessment tool evaluated by Custodio-Lumsden CL et al. [14], demonstrated the feasibility of caregiver involvement in the early detection of caries, most studies did not report measures of internal consistency, ROC analysis, optimal cutoff values, or agreement statistics. In contrast, this study assessed reliability using Cronbach’s alpha, identified an optimal diagnostic threshold through ROC analysis, and evaluated screening performance using sensitivity, specificity, predictive values, likelihood ratios, and Cohen’s kappa. These additional analyses provided stronger evidence regarding the validity and practical utility of the questionnaire as a screening instrument for ECC.

From a clinical perspective, this questionnaire may serve as an efficient and low-cost screening tool for identifying children who require early dental evaluation. Its simplicity, short administration time, and non-invasive nature make it particularly suitable for large-scale community screening programs and preventive oral health initiatives. Early identification of children at risk may facilitate timely preventive interventions, parental counseling, dietary modification, fluoride application, and referral for professional dental care, thereby reducing the burden of untreated ECC.

The present study had certain limitations. The cross-sectional design prevented the assessment of the predictive ability of the questionnaire over time. The study was conducted at a single center, which may limit the generalizability of the findings to broader populations. In addition, caregiver responses were self-reported and therefore subject to recall and social desirability biases. Although the questionnaire demonstrated good psychometric properties, external validation in different populations has not been performed. Furthermore, because participants were recruited from a single institution using a hospital-based sampling approach, selection bias cannot be completely excluded. Consequently, the study population may not fully represent the broader preschool population, and caution should be exercised when generalizing the findings to other settings or populations.

Furthermore, the questionnaire was developed, and its optimal diagnostic cutoff was derived and evaluated, using the same study population, which may have resulted in optimism bias and a slight overestimation of its diagnostic performance. External validation in independent populations was not performed and is therefore required before the questionnaire can be recommended for widespread clinical or community use. Additionally, as the study was conducted at a single center among a specific population, the findings may not be directly generalizable to populations with different demographic, socioeconomic, or cultural characteristics. Future multicenter studies involving diverse populations are warranted to externally validate the questionnaire and confirm its applicability across different settings.

Future studies should evaluate this questionnaire in larger, multicenter populations with diverse socioeconomic and cultural backgrounds. Longitudinal investigations are required to determine its predictive value for future caries development. Further refinement of the questionnaire items, incorporation into digital or mobile-based screening platforms, and comparison with established ECC risk assessment tools may enhance its applicability and clinical utility. Such efforts may contribute to the development of accessible and reliable community-based strategies for the early detection and prevention of caries in early childhood.

## Conclusions

The caregiver-completed early childhood caries risk questionnaire demonstrated good reliability, substantial agreement with the clinical diagnosis, and excellent overall screening performance. The questionnaire demonstrated high sensitivity, specificity, and overall diagnostic accuracy in identifying children with ECC. Its simple, non-invasive, and cost-effective nature makes it a practical tool for community-based screening and early referral. The incorporation of such caregiver-based assessment methods may facilitate timely preventive interventions and contribute to reducing the burden of ECC among preschool children.

## Appendices

Appendix 1

**Table 7:** Questionnaire used in the study. The questionnaire was developed by Gunnam DS, Ravikumar A, Chokshi AA, Chauhan S, Sreedhar G, and Prashanth KB.

Part A: Demographic details of study participant			
Child’s age (in years)		☐ 3 ☐ 4 ☐ 5 ☐ 6	
Child’s sex		☐ Male ☐ Female	
Relationship of respondent to child		☐ Mother ☐ Father ☐ Grandparent ☐ Other (specify):	
Caregiver’s highest education level completed		☐ No formal schooling ☐ Primary school ☐ Secondary school ☐ College/University or higher	
PART B: ECC‑related questions and instructions “Please answer the following questions about your child’s feeding, oral habits, and dental history. There are no right or wrong answers. Your honest responses will help us understand your child’s risk for tooth decay.”			
Section	Question / Item	Response options (tick)	Points
1. Breastfeeding / Bottle‑feeding at night	Does your child currently breastfeed or bottle‑feed during the night (after going to bed)?	☐ No ☐ Yes, sometimes ☐ Yes, every night or almost every night	No = 0; Sometimes = 1; Every night = 2
	If yes, does your child usually fall asleep with the breast or bottle in the mouth?	☐ No ☐ Yes	No = 0; Yes = 1
2. Use of sugary drinks and snacks	How often does your child drink sweetened liquids (milk with sugar, juice, soft drinks, flavored drinks)?	☐ Never or rarely ☐ 1 time per day ☐ 2-3 times per day ☐ More than 3 times per day	0; 1; 2; 3
	Does your child drink anything other than plain water from a bottle or sippy cup between meals or at bedtime?	☐ No, only plain water ☐ Yes, sometimes ☐ Yes, often	0; 1; 2
	How often does your child eat sweet snacks (biscuits, chocolates, candies, cakes, etc.) between meals?	☐ Less than once per day ☐ 1-2 times per day ☐ 3 or more times per day	0; 1; 2
3. Oral hygiene practices	At what age did you start cleaning or brushing your child’s teeth/gums?	☐ Before first tooth appeared / as soon as first tooth appeared ☐ After 1 year of age ☐ Not yet started / do not clean teeth	0; 1; 2
	How often are your child’s teeth brushed?	☐ Twice a day or more ☐ Once a day ☐ Less than once a day / never	0; 1; 2
	Who usually brushes the child’s teeth?	☐ Adult (parent/caregiver) brushes or supervises ☐ Child brushes alone ☐ Teeth not brushed	0; 1; 2
	What type of toothpaste is usually used for your child?	☐ Fluoridated children’s toothpaste (check the tube label) ☐ Non‑fluoridated / herbal / not sure ☐ No toothpaste used	0; 1; 2
4. Dental history and visible problems	Has your child ever visited a dentist?	☐ Yes, for routine check‑up/preventive care ☐ Yes, because of pain, holes in teeth, or infection ☐ No, never visited a dentist	Routine = 0; Pain/holes/infection = 1; No visit = 1
	Have you ever been told by a dentist or health worker that your child has tooth decay (dental caries/cavities)?	☐ No ☐ Yes ☐ Not sure	No = 0; Yes = 2; Not sure = 1
	Does your child currently complain of tooth pain or sensitivity (for example while eating, drinking, or at night)?	☐ No ☐ Yes, sometimes ☐ Yes, often	0; 1; 2
	Have you noticed any of the following in your child’s front teeth (upper or lower)? (tick all that apply)	☐ White or brown spots near the gumline ☐ Small holes or broken parts of the tooth ☐ Swelling or pus near the tooth ☐ None of the above	Spots = 1; Small holes = 2; Swelling/pus = 3; None = 0
5. Family and caregiver factors	Have you or your child’s other parent/caregiver had tooth decay in the last 1-2 years?	☐ No ☐ Yes ☐ Not sure	No = 0; Yes = 2; Not sure = 1
	How would you rate your child’s overall oral health?	☐ Excellent / very good ☐ Good ☐ Fair / poor	Excellent/very good = 0; Good = 1; Fair/poor = 2
Scoring summary	Minimum score	0	
	Maximum score	30	
